# Labour and delivery ward register data availability, quality, and utility - Every Newborn - birth indicators research tracking in hospitals (EN-BIRTH) study baseline analysis in three countries

**DOI:** 10.1186/s12913-020-5028-7

**Published:** 2020-08-12

**Authors:** Louise Tina Day, Georgia R. Gore-Langton, Ahmed Ehsanur Rahman, Omkar Basnet, Josephine Shabani, Tazeen Tahsina, Asmita Poudel, Kizito Shirima, Shafiqul Ameen, Ashish K.C., Nahya Salim, Sojib Bin Zaman, Donat Shamba, Hannah Blencowe, Harriet Ruysen, Shams El Arifeen, Dorothy Boggs, Vladimir S. Gordeev, Qazi Sadeq-ur Rahman, Tanvir Hossain, Elisha Joshi, Sabu Thapa, Rajendra Prasad Poudel, Durga Poudel, Priyanka Chaudhary, Rabina Karki, Bibek Chitrakar, Namala Mkopi, Anna Wisiko, Alodear Patrick Kitende, Marystella Revocatus Shirati, Christostomus Chingalo, Amina Omari Semhando, Cleopatra Mtei, Victoria Mwenisongole, John Mathias Bakuza, Japhet Kombo, Godfrey Mbaruku, Joy E. Lawn

**Affiliations:** 1 Maternal, Adolescent, Reproductive & Child Health (MARCH) Centre, London School of Hygiene and Tropical Medicine, London, UK; 2 Maternal and Child Health Division, International Centre for Diarrhoeal Disease Research, Bangladesh (icddr,b), Dhaka, Bangladesh; 3 Golden Community, Lalitpur, Nepal; 4 Department of Health Systems, Impact Evaluation and Policy, Ifakara Health Institute, Dar es Salaam, Tanzania; 5 Department of Women’s and Children’s Health, Uppsala University, Uppsala, Sweden; 6 Muhimbili University of Health and Allied Sciences (MUHAS), Dar es Salaam, Tanzania; 7 Institute of Population Health Sciences, Queen Mary University of London, Mile End Road, London, E1 4NS UK; 8 LifeLine Nepal, Baneshwor, Kathmandu, Nepal; 9 Yagiten Pvt Ltd, Kathmandu, Nepal; 10 Muhimbili National Hospital (MNH), Dar es Salaam, Tanzania; 11 Temeke Regional Referral Hospital, Dar es Salaam, Tanzania

**Keywords:** Maternal, Newborn, Stillbirth, Registers, Birth, Hospital, Routine Health Management Information Systems, Measurement, Indicators

## Abstract

**Background:**

Countries with the highest burden of maternal and newborn deaths and stillbirths often have little information on these deaths. Since over 81% of births worldwide now occur in facilities, using routine facility data could reduce this data gap. We assessed the availability, quality, and utility of routine labour and delivery ward register data in five hospitals in Bangladesh, Nepal, and Tanzania. This paper forms the baseline register assessment for the *Every Newborn*-Birth Indicators Research Tracking in Hospitals (EN-BIRTH) study.

**Methods:**

We extracted 21 data elements from routine hospital labour ward registers, useful to calculate selected maternal and newborn health (MNH) indicators. The study sites were five public hospitals during a one-year period (2016–17). We measured 1) availability: completeness of data elements by register design, 2) data quality: implausibility, internal consistency, and heaping of birthweight and explored 3) utility by calculating selected MNH indicators using the available data.

**Results:**

Data were extracted for 20,075 births. Register design was different between the five hospitals with 10–17 of the 21 selected MNH data elements available. More data were available for health outcomes than interventions. Nearly all available data elements were > 95% complete in four of the five hospitals and implausible values were rare. Data elements captured in specific columns were 85.2% highly complete compared to 25.0% captured in non-specific columns. Birthweight data were less complete for stillbirths than live births at two hospitals, and significant heaping was found in all sites, especially at 2500g and 3000g. All five hospitals recorded count data required to calculate impact indicators including; stillbirth rate, low birthweight rate, Caesarean section rate, and mortality rates.

**Conclusions:**

Data needed to calculate MNH indicators are mostly available and highly complete in EN-BIRTH study hospital routine labour ward registers in Bangladesh, Nepal and Tanzania. Register designs need to include interventions for coverage measurement. There is potential to improve data quality if Health Management Information Systems utilization with feedback loops can be strengthened. Routine health facility data could contribute to reduce the coverage and impact data gap around the time of birth.

## Background

Improving quality of care at birth could save an estimated 3 million lives per year [1, 2]. To drive progress, accurate data are essential, however, the majority of deaths around the time of birth occur in settings with the least information on these deaths, the “inverse data law” [3]. Improving impact and coverage data for action is central to the Sustainable Development Goal (SDG) aspiration of “no-one left behind” [4], the United Nation’s Global Strategy for Women’s Children’s and Adolescents’ Health [5], and The *Every Newborn* Action Plan (ENAP). One of five ENAP strategic objectives is to transform metrics and use of data to improve outcomes and track progress towards ending preventable maternal and newborn deaths, including stillbirths [6].

Labour and Delivery (L&D) ward registers are routinely completed by facility health workers and used to track ward admissions and discharges in a parallel system to patient case notes. Birth outcomes, care and interventions for women and babies are also often documented in these registers. However, concerns of poor register data quality in low- and middle- income counties (LMIC), have reduced confidence in full utilization of this data source in Health Management Information Systems (HMIS). As global facility births increase, currently >81%, [7], it is important to reassess the availability and quality of this routine data to help address the current data gap around the time of birth.

Research assessing labour ward register data in LMICs provides some explanation for the scepticism surrounding programmatic use of this source. Maternal and newborn health (MNH) data elements were not consistently available in facility registers in 24 high burden countries [8]. In a rural primary health care context in north eastern Nigeria health workers documented in labour ward registers most completely for birthweight (99%) and woman’s age at delivery (97%); documentation was less complete for the composite indicator essential newborn care (82%) and preterm birth (77%) [9]. In two rural Kenyan hospitals, entire labour ward registers were missing for months, and when present many data elements were less than 80% complete; the proportion of data legible/correctly coded/appropriate/recognized ranged from 29 to 100% [10]. In one Ethiopian hospital, among the 20% of births missing from the labour ward register, 91% had received a clinical intervention, thus the register both underestimated total births and interventions [11]. However, the picture is not wholly negative and routine data can be improved. Data quality improvement efforts across 20 L&D wards in South Africa, including data collection training and monthly data reviews, demonstrated increased completeness from 26 to 64%, and accuracy from 37 to 65% [12]. In Rwanda, health system strengthening measures including performance review feedback activities, mentoring, and enhanced supervision led to increased value and ownership of data among health workers [13]. In Zanzibar, quarterly data use workshops with active engagement of data users, grew and improved the HMIS, enhancing staff capacity for information use, presentation and analysis for decision making [14].

**Fig. 1:**
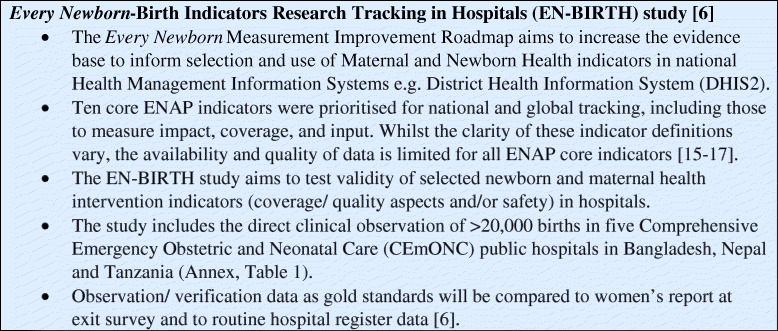
Summary of the EN-BIRTH study [6, 15–17]

The *Every Newborn*-Birth Indicators Research Tracking in Hospitals (EN-BIRTH) study aimed to assess the validity of selected newborn and maternal health care interventions indicators (coverage, content/quality, and/or safety) in hospitals [6] (Fig. 1). Our assessment of existing routine registers in EN-BIRTH study hospitals formed the baseline against which to evaluate any changes in documentation resulting from the presence of researchers in the L&D ward [18].

### Aim

This study aimed to assess the availability, quality, and utility of routine data in labour ward registers in five hospitals for 1 year before EN-BIRTH data collection.

### Objectives

To evaluate routine hospital labour ward registers for 21 selected maternal and newborn data elements (Annex, Table 2):

1. Data availability: measure completeness in relation to register design.
2. Data quality: assess implausibility, internal consistency, and birthweight heaping.
3. Data utility: cross-tabulate and transform available count data to coverage/impact indicators.

## Methods

### Study settings

The five EN-BIRTH study sites are public hospitals in high burden Sub-Saharan African and South Asian LMIC settings and implementing the selected MNH interventions. Two hospitals in Bangladesh (BD) - Maternal and Child Health Training Institute (MCHTI) Azimpur, and Kushtia District Hospital; one in Nepal (NP) - Pokhara Academy of Health Sciences; and two in Tanzania (TZ) - Temeke Regional Hospital and Muhimbili National Hospital (Annex, Table 1) [6].

### Data collection

Data elements/ count data required to calculate selected priority global MNH indicators were identified (*n* = 21) (Annex, Table 2). The data elements were extracted from routine hospital labour ward registers by trained researchers. In Bangladesh, for Caesarean section births, additional data from routine “Operation Registers” were extracted and included in the dataset. All data were extracted at the end of the 12 month study period, prior to EN-BIRTH observational data collection; in Tanzania and Bangladesh, 1st January 2016 – 31st December 2016, and in Nepal 1st April 2016 - 31st March 2017. Data were extracted for all births in Bangladesh and Nepal, and a 20% simple random monthly sample in Tanzania, due to the high case volume. Data were directly entered into customized databases in Tanzania and Nepal and into Microsoft Excel (Version 2007) from register photographs in Bangladesh.

### Data analysis

Data were analyzed in Stata 15 (StataCorp, 2017, College Station, TX).

The following data analysis methods were applied for each study objective (Table 1):

**Table 1:** Terms and definitions of data availability, quality and utility assessed by study objectives. EN-BIRTH Baseline Register Analysis

Study Objective	Term	Definition
**Objective 1: Data Availability**	**Availability**	A measure of whether the specific data element is recorded in the register in relation to register design [8, 19]
	**Completeness**	A measure of the proportion of entries in the register that had any data recorded for the specified data element for: Numerator – women or babies for whom intervention received/not received or health outcome of interest recorded Denominator – mothers delivered or babies born [20].
**Objective 2: Data Quality**	**Implausibility**	A measure of whether individual data are outside pre-defined ranges of biological credibility.
	**Heaping**	A measure of the proportion of values falling on specific values (e.g. for birthweight on 2000g or 2500g) or rounded (i.e. ending in “00” or “50”).
	**Internal consistency**	A measure of whether the observed relationship between related data elements is as expected [20, 21].
**Objective 3: Data Utilization**	**Utility**	The transformation of count data into indicators by using them as numerators and denominators or cross-tabulation.
	**Coverage**	Number of individuals receiving an intervention or service (numerator), from among the hospital population in need of the intervention or service (denominator) [6].
	**Impact**	A measure of the extent to which health status of the facility target population is being achieved (e.g. maternal and newborn mortality); used for global tracking [22] .

- **Objective 1: availability of labour ward register data elements**

*Availability of data elements:* mapped across the five hospital registers by classifying the register design into one of three categories:

- **Specific column allotted for data element** e.g. Column title: “Uterotonic for third stage of labour”, documentation requires “Yes” or “No”.

- **Non-specific column allotted for data element** e.g. Column title: “Drugs given”. Uterotonic drugs are documented alongside other drugs e.g. analgesics, antibiotics etc.

- **No column allotted** for the data element in the routine register (but may be recorded elsewhere e.g. patient case notes).

*Completeness of data element recording:* the percentage of total births recorded in the register with data recorded for the data element (Table 1). Whilst data completeness is often considered a data quality dimension, for the purpose of this study, we consider it separately [20].

- **Objective 2: quality of labour ward register data**

Three facets of data quality were assessed for a subset of data elements:

*Implausibility:* The proportion of extreme or unlikely values were calculated for three data elements: birthweight (< 350g or > 6000g), gestational age (< 20 weeks or > 44 weeks), and women’s age (< 10 years or > 49 years).

*Birthweight heaping and rounding* were assessed in three ways. First, the proportion of birthweights rounded to 100g (ending “00”) or 50g (ending “50”) was calculated.

Second, rounded weight values (e.g. 2500g) were calculated as a proportion of all weights within the adjacent 250g brackets (e.g. 2250-2750g). Third, the heaping ratio of the rounded weight value (e.g. 2500g) relative to the number of weights within the adjacent 250g brackets, excluding the rounded value (e.g. 2250–2499 plus 2501-2749g) was calculated.

*Internal consistency* of data elements with expected associations were examined by cross tabulation [23]: birth outcome and breastfeeding and [1] baby outcome at discharge [20, 24].

- **Objective 3: utility**

To explore potential use of available MNH data elements, indicators (coverage, impact, and others of programmatic relevance) (Annex Table 3) were calculated with 95% confidence intervals (95% CI) using the register count data as numerators and denominators (Annex Table 4). For indicators using live births as the denominator, our calculations include only recorded live births in both numerator and denominator. Birth outcomes were further disaggregated by birthweight [6]. The effect of birthweight heaping on the Low Birth Weight (LBW) rate was explored by reallocating 50% of the birthweights recorded as exactly 2500g to the LBW (<2500g) category.

### Ethical approval

Institutional review boards in all sites, and at the London School of Hygiene and Tropical Medicine granted ethical approval and administrative data sharing agreements were in place.

## Results

### Objective 1: availability of data

Data were extracted for 20,075 babies in total, 8544 in Nepal, 7111 in Bangladesh, and 4420 in Tanzania (Table 2). Across the five hospitals, 396 babies were either twins or triplets.

**Table 2:** Availability of data in labour ward/ operation theatre registers in five EN-BIRTH study hospitals at baseline, total births recorded *n*=20,075

	Bangladesh				Nepal	Tanzania		Total
	Azimpur Tertiary		Kushtia District		Pokhara Regional	Temeke Regional	Muhimbili National	
Register Name	Labour Ward Register	Operation Register	Labour Ward Register	Operation Register	Obstetric Register	HMIS Labour Ward Register	HMIS Labour Ward Register & Midwifery Book	
**Total number of babies extracted in register**	1415	3253	1742	701	8544	2560	1860	20,075
**Babies of multiple births (twins, triplets)**	26	60	93	6	76	121	14	396
**Total data elements in register**	18	21	19	21	31	43	45	
**Total data elements of 21 requested**	10		11		12	15	17	

The labour ward registers were named: “Delivery Register” in Azimpur BD which differed from “Delivery Register” in Kushtia, BD. Both Bangladesh hospitals used “Operation Registers” for Caesarean births (Table 2). “Obstetric Register” is the national standardized register in Pokhara NP. Both Tanzanian hospitals use the national standardized HMIS labour ward register and additional data elements are captured in Muhimbili within a informal perinatal register known locally as “Midwifery Book”.

**Fig. 2:**
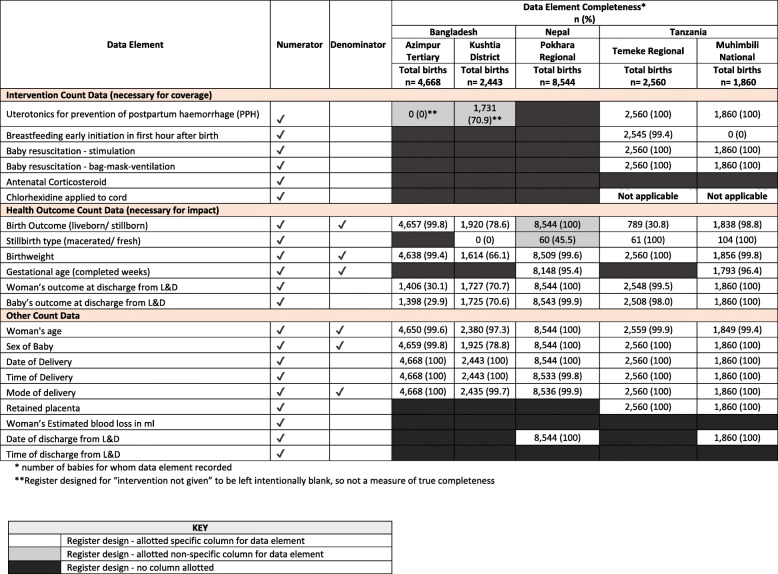
Availability and completeness of data elements in labour ward registers, by intervention, health outcome and other count data coded by register design. EN-BIRTH Baseline Register Analysis n=20,075

The labour ward register designs are summarized in Fig. 2, shaded in black if the data element is not captured. Labour ward registers contained ten of 21 data elements in Azimpur BD, 11 in Kushtia BD, 15 in Temeke TZ, 17 in Muhimbili TZ, and 12 in Pokhara NP (Table 2).

Across the five hospital registers, these 21 data elements were recorded in 65 separate columns, of which 61 columns were “specific” for the data element and four columns were “non-specific”. High completeness (>80%) was found for 85.2% of the 61 specific columns compared to 25.0% of the four non-specific colums.

#### Availability of intervention count data

Uterotonics for prevention of postpartum haemorrhage (PPH) was not captured in the register in Pokhara NP. In both the Bangladesh hospital registers uterotonics were recorded in a non-specific column “medicine given”, left blank for “not given”, so true completeness could not be calculated. The Tanzanian register had a specific column headed “Oxytocin, Ergometrine, or Misoprostol” and completeness was 100% in both Muhimbili and Temeke TZ (Fig. 2).

Immediate breastfeeding was not captured in either Bangladesh or Nepal registers. The Temeke TZ register had 99.4% completeness, but the same data element in Muhimbili TZ was not completed.

Newborn resuscitation data [25] were also only recorded in the Tanzanian registers, within a specific column “Helping Babies Breathe” coded: “1” suction, “2” stimulation, “3” bag-mask-ventilation and "no" for no resuscitation. Completeness in both hospitals was 100%.

#### Availability of health outcome data

The baby’s outcome at birth, live birth or stillborn, was documented in a non-specific column in Pokhara and a specific column in the other four hospitals. Completeness of recording was 30.8% in Temeke TZ, 78.6% in Kushtia BD and above 98.0%, for the remaining hospitals (Fig. 2).

Data elements for stillbirth (SB) timing (antepartum/ intrapartum) were not available in any register and proxy measures (fresh/macerated) were allotted a specific column in Kushtia BD (completeness 0%) and both Tanzanian hospitals (completeness 100%) and a non-specific column in Pokhara NP (completeness 45.5%) (Fig. 2).

**Fig. 3:**
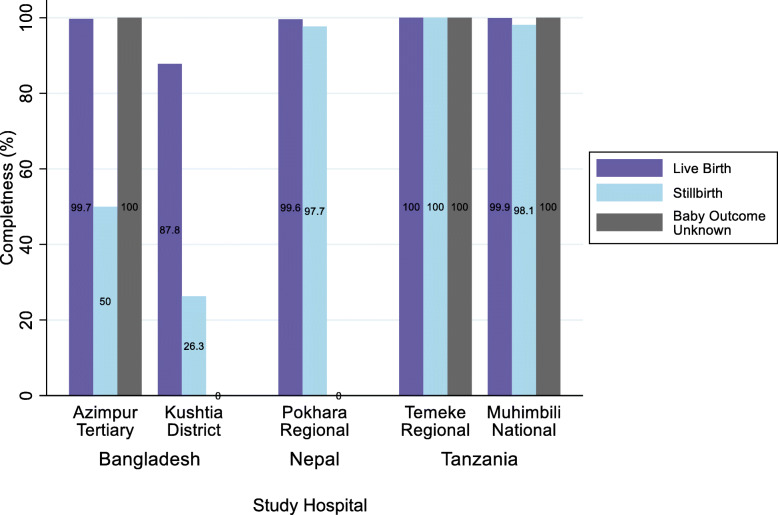
Completeness (%) of recording of birthweight data stratified by birth outcome (live birth/stillbirth/birth outcome unknown). EN-BIRTH Baseline Register Analysis n=19,177

Birthweight was documented in a specific column in all five registers, completeness was >99% in four hospitals and 66.1% in Kushtia BD. Stratifying birthweight completeness by outcome showed that in Bangladesh stillbirths were much less complete, 50.0% compared to 100% for live births in Azimpur BD and 26.3% compared to 87.8% for live births in Kushtia BD (Fig. 3). Gestational age was allotted a specific column only in Pokhara NP and in the additional perinatal register in Muhimbili TZ, completeness was >95% in both. Women’s and baby’s condition at discharge from the L&D ward had specific columns in all registers, completeness was <80% in Azimpur BD and Kushtia BD and >99.5% in Muhimbili and Temeke TZ and Pokhara NP (Fig. 2).

#### Availability of other count data

All five labour ward registers, had specific columns and >95% completeness for: woman’s age, date and time of birth, and mode of birth (Fig. 2). Baby's sex was >95% complete in all registers except Kushtia BD, 78.8%.

Antenatal corticosteroids, chlorhexidine application to cord (implemented in BD and NP), and time of discharge from L&D ward were not allotted columns in any register. Date of discharge from L&D ward was only allotted a specific column in Pokhara NP and Muhimbili TZ, 100% complete (Fig. 2).

### Objective 2: quality of data

#### Implausibility

The proportion of implausible values was low across hospitals – for birthweight 0–1.2%, for gestational age 0–0.2%, and woman’s age 0–0.2%.

#### Heaping

**Fig. 4:**
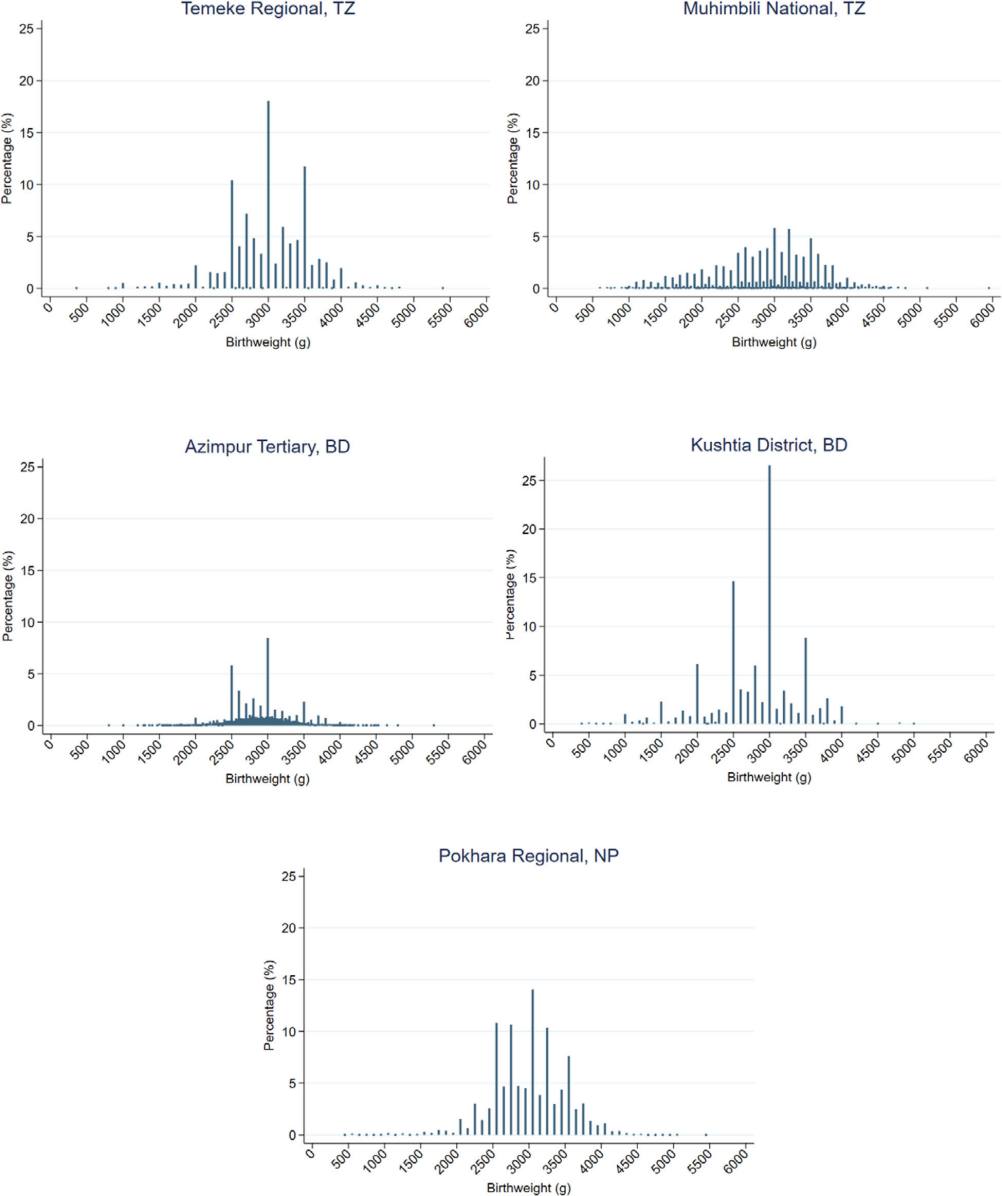
Distribution of plausible birthweights recorded in each of the five EN-BIRTH study hospital labour ward registers. EN-BIRTH Baseline Register Analysis, n = 19,140

Birthweight data were heaped in all five hospitals, in four registers more than 74% of weights were rounded to the nearest 100g (Fig. 4, Annex, Table 5). The heaping ratio was highest at 2.00 in Kushtia for 3000g, i.e. twice as many babies were recorded as exactly 3000g than at any other weight within the two adjacent 250g brackets (2750–2999 and 3000-3249g) (Annex, Table 5). For the critical 2500g LBW cut-off weight, among all babies with a birthweight within range 2250-2749g, the babies with birthweight recorded as exactly 2500g was very high; 60.7% in Kushtia BD, 43.5% in Pokhara NP, 42.0% in Temeke TZ, 19.5% in Azimpur BD and 18.9% in Muhimbili TZ (Annex, Table 5).

#### Internal consistency

Babies with birth outcome “stillbirth” should also be recorded “died” for baby outcome at L&D ward discharge. In Bangladesh, the non-specific discharge term “unwell” was recorded for 96.2% stillbirths (*n* = 25) in Azimpur and 94.6% (*n* = 106) in Kushtia. The discharge term “alive” or “well” was used for 5.4% (*n* = 6) in Kushtia BD, 16.3% (*n* = 17) in Muhimbili TZ and 6.6% (*n* = 4) in Temeke TZ. Stillbirths recorded as having been breastfed were 11.5% (*n* = 7) in Temeke TZ.

### Objective 3: utilization of data

#### Intervention coverage indicators

Coverage indicators calculated from the available register count data are shown in Table 3. Uterotonics coverage to prevent PPH ranged from 19.5% of live births in Temeke TZ to 89.1% of live births in Kushtia BD.

**Table 3:**
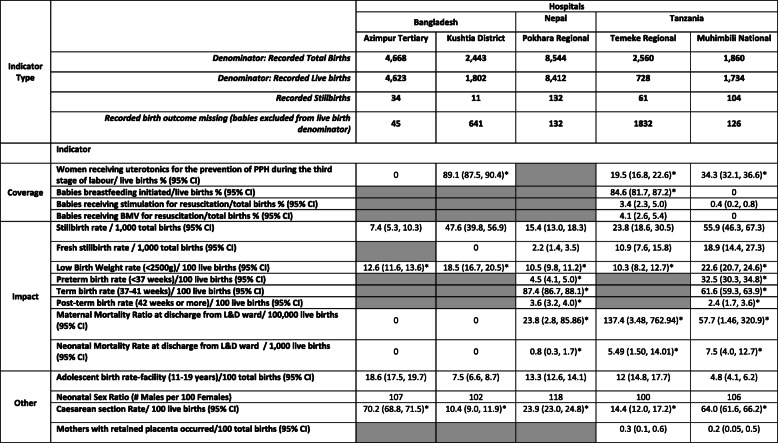
Examples of data utilization - transformation of count data into indicators - EN-BIRTH registers baseline analysis n = 20,075

* For indicators which use live births as the denominator: calculations include only live births in the numerator given the incomplete recording of birth outcome data (denominator) in all facilities

Grey cells indicate data element required to calculate indicator not present in the Labour Ward Register

The neonatal resuscitation coverage true denominator is “babies in need of resuscitation”, and as this was not available in these routine registers, a surrogate of total births (live births plus stillbirths) was used. Bag-mask ventilation (BMV) was received by 4.1% (*n* = 105) of total births in Temeke TZ (Table 3), among these 25.7% (*n* = 27) were live births, 1.9% (n = 2) were fresh stillbirths, and 72.4% (*n* = 74) had birth outcome missing. Among babies receiving BMV in Temeke TZ only 24.8% (*n* = 26) were recorded to have also received stimulation.

#### Impact indicators

The facility stillbirth rate (SBR) was lowest at 7.4 in Azimpur BD and highest at 55.9 in Muhimbili TZ per 1000 total births, Table 3. The fresh SBR ranged from 2.2 in Pokhara NP to 18.9 in Muhimbili TZ per 1000 total births.

Low Birth Weight (LBW) prevalence, ranged from 10.3% in Temeke TZ to 22.6% in Muhimbili TZ, Table 3. The adjusted LBW rate (after re-allocating 50% of babies with a recorded birthweight of exactly 2500g to the LBW category) increased the LBW prevalence by 1.7% in Muhimbili TZ and by 7.2% in Kushtia BD (Table 4).

**Table 4:** Adjusted and unadjusted Low Birth Weight rate - EN-BIRTH register baseline analysis n=17,033

	Bangladesh		Nepal	Tanzania	
	Azimpur Tertiary	Kushtia District	Pokhara Regional	Temeke Regional	Muhimbili National
**Unadjusted Low Birth Weight rate (< 2500 g) / 100 live births (95% CI)**	12.6 (11.6, 13.6)	18.5 (16.7, 20.5)	10.5 (9.8, 11.2)	10.3 (8.2, 12.7)	22.6 (20.7, 24.6)
**Adjusted Low Birth Weight rate (< 2500 g) / 100 live births (95% CI)**	15.6 (14.5, 16.6)	25.7 (23.6, 27.9)	15.9 (15.2, 16.7)	15.0 (12.6, 17.8)	24.4 (22.4, 26.4)
**Increase in Low Birth Weight rate (< 2500 g)**	2.93	7.20	5.41	4.67	1.73

Cross-tabulating categorical birthweight with outcome (live birth/ fresh stillbirth/ macerated stillbirth) showed 62.4% (*n* = 212) of total stillbirths and 49.3% (*n* = 41) of fresh stillbirths were categorised LBW compared to 13.1% (*n* = 2225) of live births (Table 5).

**Table 5:** Birth outcomes cross-tabulated by categorical birthweight, pooled data all EN-BIRTH hospitals baseline register analysis n=17,595

Birth Outcome	Total Births^**a**^	Categorical Birthweight (g) n (%)				
		≤999g	1000–1999g	2000–2499g	2500–3999g	≥4000g
**Total Live births (%)**	17,033	27 (0.2)	508 (3.0)	1690 (9.9)	14,402 (84.6)	361 (2.4)
**Total Stillbirths (%)**^**b**^	340	29 (8.5)	125 (36.8)	58 (17.1)	119 (35.0)	9 (2.6)
**Fresh Stillbirths (%)**^**c**^	83	4 (4.8)	27 (32.5)	10 (12.0)	39 (47.0)	3 (3.6)
**Macerated Stillbirths (%)**^**2**^	139	11 (7.9)	66 (47.5)	24 (17.3)	36 (25.9)	2 (1.4)

^a^babies with a recorded birth outcome and a plausible birthweight recorded (*n* = 17,595)

^b^includes all stillbirths from all five hospitals, ^c^Information on fresh or macerated stillbirths presented where available (i.e. for 100% of SB in Tanzania, 45.5% of SB in Nepal and no SB in Bangladesh)

The preterm birth rate (number of babies < 37 weeks per 100 live births) was 4.5% in Pokhara NP and 32.5% in Muhimbili TZ.

Maternal deaths were recorded in Pokhara NP (*n* = 3), Muhimbili TZ (*n* = 1), and Temeke TZ (*n* = 5), with none in Azimpur BD or Kushtia BD. Thus facility Maternal mortality ratio (MMR) before discharge from L&D ward ranged from zero in both Bangladesh hospitals to 137.4 per 100,000 live births in Temeke TZ (Table 3). The neonatal mortality rate (NMR) before discharge from L&D ward ranged from zero in Azimpur BD and Kushtia BD to 7.5 per 1000 live births in Muhimbili TZ.

#### Other indicators of programmatic relevance

The proportion of hospital births to adolescents (11–19 years) ranged from 4.8% in Muhimbili TZ to 18.6% in Azimpur BD. Ratio of male:female babies was highest in Pokhara NP at 118:100, Table 3.

Caesarean section rate, using a live birth denominator [26, 27], was 43.4%, ranging from 10.4% in Kushtia to 70.2% in Azimpur BD, Table 3. As 69 stillbirths (20.2% of total stillbirths) were also delivered by Caesarean, if these were included in the denominator [27], the Caesarean rate would decrease overall to 37.3%.

## Discussion

This is the largest multi-country study we are aware of in LMICs to assess labour ward register data availability, quality, and utility. Hospital registers are key tools used to collect individual data for aggregation and transmission up the HMIS data pyramid [6]. Data extracted from five CEmONC hospitals show that a large amount of data are being collected in labour ward registers. The calculation of MNH coverage and impact indicators require the availability of specific data elements for use as numerators and/or denominators, yet none of the labour ward registers contained all 21 selected data elements. Data for outputs, outcomes, and impact measurement were more widely available, than for intervention coverage. Only the Tanzanian registers captured most of the selected interventions and gestational age was only captured by the Nepal register and the additional register in Muhimbili TZ.

The Performance of Routine Information System Management (PRISM) framework identifies complexity and design as technical factors in routine health information systems performance [28]. The register designs were different between countries, and within country in Bangladesh. Data were captured from the additional perinatal register in Muhimbili TZ and from operation registers for babies born by Caesarean in Bangladesh, highlighting further complexity in multiple recording systems.

Whether a specific column was allotted for the data element related to completeness of recording. Across all five hospitals, a much lower proportion of non-specific columns had high levels of completeness than did specific columns (25.0% versus 85.2%). However, there were other examples of low completeness within specific columns (e.g. 0% SB type Kushtia BD) or high completeness within non-specific columns (e.g. 100% birth outcome, Pokhara NP) highlighting that technical factors alone are necessary but not sufficient for data availability.

Other factors associated with data completeness included mode of birth, e.g. in Kushtia BD 97.1% of babies for whom birth outcome was missing were delivered by Caesarean. This finding is similar to previous research in Ethiopia, where a high proportion of babies not recorded in the register had required a clinical intervention [11]. Previous studies have highlighted the low value placed on stillbirths and the resultant data gaps [29–31] and similarly we found birthweight data completeness was lower for these babies in Bangladesh.

Incomplete count data affect indicator calculation results. When intervention coverage numerators are missing, rates will appear low, e.g. when only the outcome “unwell” was recorded and no maternal/ newborn deaths, the MMR, NMR at discharge from L&D ward and SBR may be inappropriately low - zero in Azimpur BD and Kushtia BD during this study. When denominators such as birth outcome are missing (e.g. for 69.2% of babies in Temeke TZ and 21.4% in Kushtia BD) many indicators which use live birth as the denominator will be adversely affected. For example, coverage of breastfeeding in Temeke TZ would be 292.5% had the numerator not been restricted to include only babies in the live births denominator (i.e. exluding babies with a birth outcome unknown). Calculating coverage using a total birth denominator instead of a clinical need denominator, as we have done for neonatal resuscitation, requires benchmarked rates for meaningful tracking across hospitals.

Alternatively, data completeness may be high, but if inaccurate, coverage will be falsely low or high. For example, uterotonic coverage was apparently low in the Tanzanian registers. These data are handwritten using a Swahili abbreviation “N” for “Ndiyo” (Yes) or “H” for “Hapana” (No) which can be hard to distinguish and possibly incorrectly extracted. Numbered coding systems may be helpful when the design is simple e.g. the “Helping Babies Breathe” column in Tanzania. Blank data elements in the register can mean either “incomplete” or a true zero, as in the Bangladesh register design, which if not differentiated can introduce another source of data inaccuracy [32].

Beyond data completeness, our data quality evaluation showed variable results. Birthweight rounding and heaping were substantial across all hospitals. If a baby whose true birthweight of 2470g is rounded to 2500g the LBW rate will be underestimated – in our model by up to 7.2%. Both analogue and digital scales were used for birthweight across the five hospitals which may contribute to rounding. Additional EN-BIRTH analyses are exploring accuracy and processes of birthweight measurement [33, 34]. In these high mortality burden countries, very large variation in hospital mortality rates may suggest data quality issues; Muhimbili TZ had a stillbirth rate almost eight times higher that Azimpur BD. The EN-BIRTH mixed-methods study aims to test validity of these indicator measurements against the gold standard of observation data.

Barriers and enablers to recording in routine hospital registers are being explored in the wider EN-BIRTH study [6]. Quality of register data is affected by HMIS input determinants described by the PRISM framework [28] including technical, organisational and behavioural factors. Factors known to negatively impact routine data quality include poor use of data, lack of feedback, low management support, lack of health worker confidence, low motivation, lack of competence and low perceived utility of routine recording tasks [14, 28, 35]. Health worker training and supportive supervision regarding the importance of routine recording around the time of birth could improve data quality for all babies, especially stillbirths. Innovations to increase health worker data utilization skills could also help sustain improvements in data recording as the purpose of these activities is recognised. Previous studies have demonstrated large gains from such efforts [12–14, 35, 36].

Further research is needed to understand the effect of labour ward register design on data quality, the impact of increased reporting burden on frontline health workers, and ways to optimize the utility of register data whilst reducing duplication. Standardized and harmonized registers with inclusion of an appropriate number of selected key data elements need evaluating against registers that contain large numbers of data elements. At the five study hospitals, all documentation at the health worker/ mother and baby interface was in paper-based routine registers. As electronic platforms increase, the effect of digitization on data quality around the time of birth requires attention from the source data to the top of the data pyramid [6].

Utilizing the EN-BIRTH multi-country study hospitals, a strength of this research is the large amount of data extracted (20,075 births), providing the first in-depth and multi-country analysis of routine labour ward register data. However, EN-BIRTH study hospitals may not be entirely representative of routine recording practices in facilities at different levels of the health system nor in other LMIC settings. Some EN-BIRTH hospitals have been involved in previous research, thus routine recording may be better than typical. Conversely, staff workload in these high-volume CEmONC hospitals could reduce data quality. We were unable to assess whether all babies born on the labour ward were recorded in the register, nor the relationship between staff levels and data quality. Research in facilities at different levels of the health system is required before wider conclusions can be drawn. Furthermore, evidence of completeness and quality of register data do not necessarily correlate with accurate aggregation and reporting from the facility up the HMIS data pyramid, therefore research is required to review quality of facility-reported data used for district/national/global tracking of MNH indicators.

The *Every Newborn* strategic objective to transform measurement aims to increase availability and quality of data to use for action. Unless all births occur in hospitals, facility data will overestimate population coverage. However, as hospital births increase (globally now 81% [7]) this data source is increasingly valuable. Improving facility data quality would also benefit wider health system indicators (e.g. immunization coverage) which currently use census projection data. Whilst household surveys are useful to provide information on contact with MNH services at a population level in LMICs, they have been shown to be less valid for the capture of content or coverage of interventions around the time of birth, hence new strategies incorporating multiple data sources, including register data, are required [32, 37–39]. Clarity is needed on the calculation of Caesarean section rates; the current denominator recommendation is live births, but more than 10% of stillbirths in this dataset were delivered by Caesarean [26, 27]. In our study, inclusion of stillbirths in the denominator as well as the numerator increased Caesarean rate by nearly 6%. We propose Caesarean rates be calculated using hospital total births and stratified for live births and stillbirths.

Findings presented here could be used now by decision makers at various levels of the health system. In the hospital for quality improvement e.g. if no fresh stillbirths are being resuscitated this could lead to review of guidelines, practice and/or documentation. Birthweight data were readily available in all five hospitals, so LBW rate reporting, one of WHO 100 core health indicators could be improved [40]. If the LBW rate is implausibly low, hospitals might use the same data to improve quality of birthweight measurement. Using birthweight categories and birth outcome data, we found differences between live births and stillbirths, e.g. the differential growth of stillbirths where 36.8% weighed 1000-1999 g compared to 3.0% of live births. Our study showed that 50.6% of fresh stillbirths had a normal birthweight yet died, this metric could also be tracked to improve quality of care.

Changes in register recording practices during the EN-BIRTH study will be explored [6]. Importantly, the EN-BIRTH observational study will further validate indicators from labour ward register data to inform use in HMIS and areas of focus to further improve data availability and quality.

Data used for action is foundational for tracking progress towards global goals for every woman and every child to survive and thrive [4, 5]. As data is used, data quality and overall HMIS performance improves [14, 28]. As data quality improves, coverage and outcome indicators can more confidently be used for action to track progress and drive change.

## Conclusions

**Fig. 5:**
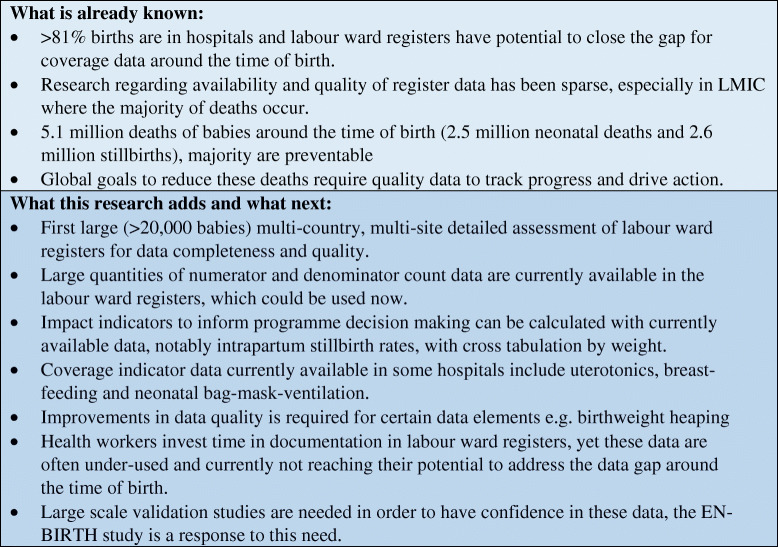
Summary figure: Labour and delivery ward register data, what is already known, what the EN-BIRTH baseline register study adds and what next

This study shows that large amounts of specific MNH data elements are currently available in routine labour ward registers in five hospitals in Bangladesh, Nepal and Tanzania. Data quality varied when assessed for completeness and implausibility. There is potential to improve the quality of available data if HMIS utilization with feedback loops can be strengthened. By advancing routine health facility data for use, labour ward registers can contribute to much needed regular coverage and impact measurements around the time of birth (Fig. 5). To optimize care around the critical time of birth, labour ward register data offer huge potential to be improved and used.

## Supplementary information

**Additional file 1: Table S1.** Summary information on country context and five EN-BIRTH study hospitals. **Table S2.** MNH data elements (*n* = 21) extracted from the labour ward and OT ward register of each of five EN-BIRTH hospitals. **Table S3.** Definitions and examples of coverage and impact indicators, including numerator and denominator. **Table S4.** Indicators calculated for potential data utilization from routine register numerator and denominatorIndicators calculated for potential data utilization from routine register numerator and denominator. **Table S5.** Heaping of birthweight data recorded labour ward/operation registers in EN-BIRTH study hospitals.

## Data Availability

The datasets used and/or analysed during the current study are available on LSHTM Data Compass repository, https://datacompass.lshtm.ac.uk/955/.

## Abbreviations

ACS: Antenatal corticosteroids
BD: Bangladesh
BMV: bag-mask ventilation
CIFF: Children’s Investment Fund Foundation
DHIS2: District Health Information Systems 2
EmONC: Emergency Obstetric and Newborn Care
ENAP: *Every Newborn* Action Plan now branded as *Every Newborn*
EN-BIRTH: *Every Newborn*-Birth Indicators Research Tracking in Hospitals study
EPMM: Ending Preventable Maternal Mortality
HMIS: Health Management Information Systems
icddr, b: International Centre for Diarrheal Disease Research, Bangladesh
IHI: Ifakara Health Institute
L&D: Labour and Delivery
LBW: Low Birth Weight
LMIC: Low and Middle Income Countries
LSHTM: London School of Hygiene and Tropical Medicine
MMR: Maternal Mortality Ratio
MNH: Maternal and Newborn Health
MUHAS: Muhimbili University of Health and Allied Sciences
NMR: Neonatal Mortality Rate
NP: Nepal
PPH: Post-partum haemorrhage
PRISM: Performance of Routine Information System Management
RHIS: Routine Health Information Systems
SB: Stillbirth
SBR: Stillbirth Rate
TZ: Tanzania
UNICEF: United Nations International Children’s Emergency Fund
WHO: World Health Organization
