# Supplementary material for: Labour and delivery ward register data availability, quality, and utility - Every Newborn - birth indicators research tracking in hospitals (EN-BIRTH) study baseline analysis in three countries
Source: BMC Health Serv Res. 2020 Aug 12;20:737. doi: 10.1186/s12913-020-5028-7 (PMC7422224; doi:10.1186/s12913-020-5028-7)
Supplement: Supplementary file 1 — Additional file 1: Table S1. Summary information on country context and five EN-BIRTH study hospitals. Table S2. MNH data elements (n = 21) extracted from the labour ward and OT ward register of each of five EN-BIRTH hospitals. Table S3. Definitions and examples of coverage and impact indicators, including numerator and denominator. Table S4. Indicators calculated for potential data utilization from routine register numerator and denominatorIndicators calculated for potential data utilization from routine register numerator and denominator. Table S5. Heaping of birthweight data recorded labour ward/operation registers in EN-BIRTH study hospitals. [file 12913_2020_5028_MOESM1_ESM.docx]

**Table S1:** Summary information on country context and five EN-BIRTH study hospitals (adapted from Day et al (1).

|  | Context | Facilities | | |
| --- | --- | --- | --- | --- |
| Country | **National mortality rates** | **Name** | **Hospital type** | **Annual Total births*** |
| Bangladesh | MMR = 176/100,000  NMR = 20/1,000  SBR = 25/1,000 | Maternal and Child Health Training Institute (MCHTI) Azimpur,  Dhaka | Tertiary | 4,668 |
|  |  | Kushtia District Hospital | District | 2,443 |
| Nepal | MMR = 258 /100,000  NMR = 22/1,000  SBR = 18/1,000 | Pokhara Academy of Health Sciences | Regional | 8,544 |
| Tanzania | MMR = 398 /100,000  NMR = 22/1,000  SBR = 22/1,000 | Temeke Regional Hospital,  Dar es Salaam | Regional | 12,800 |
|  |  | Muhimbili National Hospital,  Dar es Salaam | National Referral & University Teaching | 9,300 |
| ^*^Period 1s^t^ January 2016- 31^st^ December 2016 in Tanzania and 1^st^ April 2016 – 31^st^ March 2017 in Nepal.MMR = maternal mortality ratio per 100,000 live births (2) NMR = neonatal mortality rate per 1000 live births (3) SBR = stillbirth rate per 1000 total births (4) | | | | |

**References**

1. Day LT, Ruysen H, Gordeev VS, Gore-Langton GR, Boggs D, Cousens S, et al. “Every Newborn-BIRTH” protocol: observational study validating indicators for coverage and quality of maternal and newborn health care in Bangladesh, Nepal and Tanzania. J Glob Health. 2019;9(1):010902.
2. Trends in maternal mortality: 1990 to 2015: estimates by WHO, UNICEF, UNFPA, World Bank Group and the United Nations Population Division. Geneva: World Health Organization; 2015. Available from: <https://www.measureevaluation.org/resources/publications/ms-17-117>.
3. Blencowe H, Cousens S, Oestergaard MZ, Chou D, Moller A-B, Narwal R, et al. National, regional, and worldwide estimates of preterm birth rates in the year 2010 with time trends since 1990 for selected countries: a systematic analysis and implications. Lancet. 2012;379(9832):2162–72.
4. Blencowe H, Cousens S, Jassir FB, Say L, Chou D, Mathers C, et al. National, regional, and worldwide estimates of stillbirth rates in 2015, with trends from 2000: a systematic analysis. Lancet Glob Health. 2016;4(2):e98–e108.

**Table S2:** MNH data elements (n=21) extracted from the labour ward and OT ward register of each of five EN-BIRTH hospitals:

| 1 | Uterotonics for prevention of postpartum haemorrhage |
| --- | --- |
| 2 | Breastfeeding early initiation in first hour after birth |
| 3 | Baby resuscitation - stimulation |
| 4 | Baby resuscitation - bag-mask-ventilation |
| 5 | Antenatal Corticosteroid |
| 6 | Chlorhexidine applied to cord |
| 7 | Birth Outcome (liveborn/ stillborn) |
| 8 | Stillbirth type (antepartum/intrapartum or macerated/fresh) |
| 9 | Birth weight |
| 10 | Gestational age (completed weeks) |
| 11 | Woman’s outcome at discharge from Labour and Delivery |
| 12 | Baby’s outcome at discharge from Labour and Delivery |
| 13 | Woman’s age |
| 14 | Sex of Baby |
| 15 | Date of birth |
| 16 | Time of birth |
| 17 | Mode of birth |
| 18 | Retained placenta |
| 19 | Estimated blood loss in ml |
| 20 | Date of discharge from Labour and Delivery |
| 21 | Time of discharge from Labour and Delivery |

**Table S3:** Definitions and examples, of coverage and impact indicators, including numerator and denominator.

| **Type of indicator** | **Definition** | **Indicator examples calculated** | | |
| --- | --- | --- | --- | --- |
|  |  |  | **Numerator** | **Denominator** |
| Coverage | Number of individuals receiving an intervention or service (numerator), from among the population in need of the intervention or service (denominator)(6) | Caesarean section rate | Number of babies born by Caesarean section | Total live births |
|  |  | Early Initiation of Breastfeeding rate | Number of babies breastfed within one hour of birth | Total live births |
| Impact | A number, proportion, percentage or rate that helps measure (“indicate”) the extent to which programme achievements have been made (outcome and impact indicators) (17) | Facility Neonatal Mortality Rate | Number of neonatal deaths in facility | Total live births |
|  |  | Stillbirth Rate | Number of stillbirths | Total births (live births plus stillbirths) |
|  |  | Low birthweight rate | Number of babies with birthweight <2,500g | Births with a birthweight recorded |

**Table S4:** Indicators calculated for potential data utilization from routine register numerator and denominator.

|  | **Indicator** | **Numerator** | **Denominator** |
| --- | --- | --- | --- |
| **Coverage** | Uterotonics immediately after birth for the prevention of PPH / live births (%) | Number of women receiving uterotonics for the prevention of PPH | Total live births* |
|  | Early initiation of breastfeeding /live births (%) | Number of babies breastfed within one hour | Total live births |
|  | Stimulation for neonatal resuscitation/total births** (%) | Number ofl babies stimulated for resuscitation | Total births** |
|  | Bag-mask-ventilation for neonatal resuscitation/total births^*^ (%) | Number of babies receiving bag-mask-ventilation for resuscitation | Total births** |
| **Impact** | Stillbirth rate / 1,000 total births | Number of stillbirths | Total births |
|  | Fresh stillbirth rate / 1,000 total births | Number of fresh stillbirths | Total births |
|  | Low Birth Weight rate (<2500g)/ live births (%) | Number of babies with birthweight <2500g | Total live births |
|  | Preterm birth rate (<37 weeks)/ live births (%) | Number of babies with gestational age <37 weeks | Total live births |
|  | Term birth rate (37-41 weeks)/ live births (%) | Number of babies with gestational age 37-41 weeks | Total live births |
|  | Post-term birth rate (42 weeks or more)/ live births (95% CI)(%) | Number of babies with gestational age >42 weeks | Total live births |
|  | Facility Maternal Mortality Ratio/ 100,000 live births | Number of maternal deaths in facility | 100,000 live births |
|  | Facility Neonatal Mortality Rate / 1,000 live births | Number of neonatal deaths in facility | 1,000 live births |
| **Other** | Adolescent (11-19 years) birth rate - facility / total births (%) | Number of adolescent (11-19 years) women who gave birth in facility | Total births |
|  | Neonatal sex ratio (# males per 100 Females) | Number of male babies | Number of female babies |
|  | Caesarean section rate/ live births (%) | Number of babies born by Caesarean section | Total live births |
|  | Women with retained placenta / total births (%) | Number of women with retained placenta | Total births |
| Key: * Livebirth denominator used as per previous definition, current definition is total births denominator **Surrogate denominator as true coverage denominator of clinical need not available.  PPH = Postpartum Haemorrhage | | | |

**Table S5:** Heaping of birthweight data recorded labour ward/operation registers in EN-BIRTH study hospitals.

|  |  | **Azimpur Tertiary** | **Kushtia District** | **Pokhara Regional** | **Temeke Regional** | **Muhimbili National** | |
| --- | --- | --- | --- | --- | --- | --- | --- |
|  | **Birthweight recorded (n)** | **4,638** | **1,614** | **8,509** | **2,560** | **1,856** | |
|  |  |  |  |  |  |  | |
| **1,500g heaping** | **Exactly 1500g (n)** | 7 | 37 | 25 | 14 | 22 | |
|  | **1250-1499g/1501-1749g (n)** | 22 | 26 | 42 | 28 | 94 | |
|  | **Total 1251-1749g (n)** | 29 | 63 | 67 | 42 | 116 | |
|  | **1500g heaping ratio*** | 0.32 | 1.42 | 0.60 | 0.50 | 0.23 | |
|  | **heaped at 1500g (%)** | 24.1 | 58.7 | 37.3 | 33.3 | 19.0 | |
| **1,900g heaping** | **Exactly 1900g (n)** | 2 | 13 | 17 | 12 | 26 | |
|  | **1650-1899g/ 1901-2149g** | 125 | 144 | 253 | 81 | 150 | |
|  | **Total 1651-2149g (n)** | 127 | 157 | 270 | 93 | 176 | |
|  | **1900g heaping ratio*** | 0.02 | 0.09 | 0.07 | 0.15 | 0.17 | |
|  | **heaped at 1900g (%)** | 1.6 | 8.3 | 6.3 | 12.9 | 14.8 | |
| **2,000g heaping** | **Exactly 2000g (n)** | 35 | 99 | 126 | 57 | 34 | |
|  | **1750-1999g/ 2001-2249g** | 159 | 67 | 220 | 65 | 162 | |
|  | **Total 1751-2249g (n)** | 194 | 166 | 346 | 122 | 196 | |
|  | **2000g heaping ratio*** | 0.22 | 1.48 | 0.57 | 0.88 | 0.21 | |
|  | **heaped at 2000g (%)** | 18.0 | 59.6 | 36.4 | 46.7 | 17.4 | |
| **2,400g heaping** | **Exactly 2400g (n)** | 26 | 19 | 211 | 40 | 32 | |
|  | **2150-2399g/ 2401-2649g (n)** | 1,118 | 338 | 1,700 | 450 | 281 | |
|  | **Total 2150-2649g (n)** | 1,144 | 357 | 1,911 | 490 | 313 | |
|  | **2400g heaping ratio*** | 0.02 | 0.06 | 0.12 | 0.09 | 0.11 | |
|  | **heaped at 2400g (%)** | 2.27 | 5.32 | 11.04 | 8.16 | 10.22 | |
| **2,500g heaping** | **Exactly 2500g (n)** | 270 | 236 | 916 | 266 | 63 | |
|  | **2250-2499g/ 2501-2749g (n)** | 1,118 | 153 | 1,191 | 368 | 271 | |
|  | **Total 2250-2749g (n)** | 1,388 | 389 | 2,107 | 634 | 334 | |
|  | **2500g heaping ratio*** | 0.24 | 1.54 | 0.77 | 0.72 | 0.23 | |
|  | **heaped at 2500g (%)** | 19.5 | 60.7 | 43.5 | 42.0 | 18.9 | |
| **2,900g heaping** | **Exactly 2900g (n)** | 89 | 36 | 379 | 85 | 71 | |
|  | **2650-2899g/ 2901-3149g (n)** | 2,052 | 603 | 2,827 | 831 | 400 | |
|  | **Total 2650-3149g(n)** | 2,141 | 639 | 3,206 | 916 | 471 | |
|  | **2900g heaping ratio*** | 0.04 | 0.06 | 0.13 | 0.10 | 0.18 | |
|  | **heaped at 2900g(%)** | 4.2 | 5.6 | 11.8 | 9.3 | 15.1 | |
| **3,000g heaping** | **Exactly 3000g (n)** | 393 | 428 | 1192 | 460 | 107 | |
|  | **2750-2999g/ 3001-3249g (n)** | 1,669 | 214 | 1,659 | 422 | 433 | |
|  | **Total 2750-3249g (n)** | 2,062 | 642 | 2,851 | 882 | 540 | |
|  | **3000g heaping ratio*** | 0.24 | 2.00 | 0.72 | 1.09 | 0.25 | |
|  | **heaped at 3000g (%)** | 19.1 | 66.7 | 41.8 | 52.2 | 19.8 | |
| **3,400g heaping** | **Exactly 3400g (n)** | 45 | 18 | 365 | 119 | 56 | |
|  | **3150-3399g/ 3401-3649g (n)** | 861 | 247 | 1,995 | 622 | 416 | |
|  | **Total 3150-3649g (n)** | 906 | 265 | 2,360 | 741 | 472 | |
|  | **3400g heaping ratio*** | 0.05 | 0.07 | 0.18 | 0.19 | 0.13 | |
|  | **heaped at 3400g (%)** | 5.0 | 6.8 | 15.5 | 16.1 | 11.9 | |
| **3,500g heaping** | **Exactly 3500g (n)** | 106 | 143 | 642 | 299 | 89 | |
|  | **3250-3499g/ 3501-3749g (n)** | 599 | 93 | 1,001 | 361 | 302 | |
|  | **Total 3250-3749g (n)** | 705 | 236 | 1,643 | 660 | 391 | |
|  | **3500g heaping ratio*** | 0.18 | 1.54 | 0.64 | 0.83 | 0.29 | |
|  | **heaped at 3500g (%)** | 15.0 | 60.6 | 39.1 | 45.3 | 22.8 | |
|  | **Birthweight ends in “00” (%)** | 37.4 | 99.5 | 86.2 | 99.1 | 74.8 | |
|  | **Birthweight ends in “50” (%)** | 8.0 | 0.4 | 12.8 | 0.4 | 13.4 | |
| *** ratio of birthweights recorded as exact value (e.g. 2500g) relative to number of babies within 500g range of this value but not including value (e.g. 2250-2499g/ 2501-2749g)** | | | | | | |  |
